# Apixaban-Calibrated Anti-FXa Activity in Relation to Outcome Events and Clinical Characteristics in Patients with Atrial Fibrillation: Results from the AVERROES Trial

**DOI:** 10.1055/s-0037-1613679

**Published:** 2017-12-12

**Authors:** Vinai C. Bhagirath, John W. Eikelboom, Jack Hirsh, Michiel Coppens, Jeffrey Ginsberg, Thomas Vanassche, Fei Yuan, Noel Chan, Salim Yusuf, Stuart J. Connolly

**Affiliations:** 1 Department of Medicine, McMaster University, Hamilton, Ontario, Canada; 2 Population Health Research Institute, McMaster University, Hamilton, Ontario, Canada; 3 Department of Vascular Medicine, Academic Medical Centre, Amsterdam, The Netherlands; 4 Department of Cardiovascular Sciences, University Hospital, Leuven, Belgium

**Keywords:** apixaban, fibrillation, hemorrhage, systemic embolism

## Abstract

**Background** In patients with nonvalvular atrial fibrillation (AF), apixaban is given in doses of 5 or 2.5 mg twice daily, according to clinical characteristics. The usual on-treatment range of apixaban drug levels, as determined by apixaban-calibrated anti-factor Xa (anti-Xa) activity, has previously been measured in small cohorts; however, the association between anti-Xa activity and clinical outcomes and the predictors of variability in anti-Xa activity have not been well studied in the AF population.

**Methods and Results** Anti-Xa activity was measured before taking the morning dose, 3 months after enrollment in the AVERROES study using a calibrated anti-Xa assay (Rotachrom). Patients with two of the following criteria—age >80; weight <60 kg; or creatinine >133 μg/L—received 2.5 mg twice daily ( *n* = 145), while all others received 5 mg twice daily ( *n* = 2,247). A total of 2,392 patients were included, with median follow-up of 1.1 years. Median apixaban anti-Xa activity was 122 ng/mL (interquartile range [IQR]: 63–198 ng/mL) for the entire group; 99 ng/mL (IQR: 60–146 ng/mL) for the 2.5-mg group; and 125 ng/mL (IQR: 64–202 ng/mL) for the 5-mg group ( *p* = 0.003). A relationship was evident between bleeding and anti-Xa activity ( *p* = 0.01), which was driven by minor bleeding. No relationship was evident between major bleeding or stroke/systemic embolism and anti-Xa activity. In those receiving the 5-mg dose, estimated glomerular filtration rate, sex, and age had the strongest association with anti-Xa activity.

**Conclusion** There is considerable variability in anti-Xa activity among AF patients receiving apixaban. Rates of major bleeding and stroke/systemic embolism were low irrespective of anti-Xa activity.

**Clinical Trial Registration** ClinicalTrials.gov NCT00496769; https://clinicaltrials.gov/ct2/show/NCT00496769 .

## Introduction

Apixaban, a direct factor Xa (FXa) inhibitor, has been evaluated in two large phase III randomized controlled trials of stroke prevention in patients with atrial fibrillation (AF). 1 2 The ARISTOTLE trial demonstrated that fixed-dose unmonitored apixaban compared with dose-adjusted warfarin was associated with a similar rate of ischemic stroke, a halving of hemorrhagic stroke, and a one-third reduction in major bleeding. 1 When compared with aspirin in the AVERROES trial, fixed-dose apixaban reduced the risk of ischemic stroke by one-half, with similar rates of major bleeding and hemorrhagic stroke. 2 The effects of apixaban relative to warfarin or aspirin were consistent across all subgroups examined, including participants with advanced age, moderate renal impairment, or prior stroke. 3 4 5 6

In both trials, apixaban was administered as 5 mg twice daily unless patients met criteria (two or more of age ≥ 80 years, weight <60 kg, and serum creatinine > 1.5 mg/dL) for reduced dose apixaban 2.5 mg twice daily. This dosing strategy was associated with low rates of stroke (0.97 and 1.4%/year) and bleeding (1.4 and 2.13%/year) in the ARISTOTLE and AVERROES trials, respectively, suggesting that any possible incremental benefit of dose adjustment based on the results of routine drug level monitoring would be marginal. On the other hand, measurement of apixaban levels may be useful in selected situations. These include the management of patients who present with bleeding or acute stroke and in those with clinical characteristics that are associated with a risk of extreme drug levels. In such situations, knowledge of expected drug levels, the relationship between drug levels and clinical events, and determinants of drug levels can help guide clinical decision making.

In the AVERROES pharmacokinetic substudy, apixaban-calibrated anti-Xa activity (hereafter referred to as anti-Xa activity) was measured at steady state immediately prior to the morning dose of study drug. Our objectives were (1) to establish the range of usual on-treatment trough apixaban anti-Xa activity; (2) to examine the relationship between trough anti-Xa activity and bleeding and thromboembolic events; and (3) to explore the contribution of various clinical characteristics to apixaban anti-Xa activity.

## Materials and Methods

### Trial Design, Outcome Definitions, and Blood Sample Collection

The AVERROES trial 2 was designed to determine whether apixaban is superior to aspirin for stroke prevention in patients with AF and an additional risk factor for stroke (CHADS _2_ score ≥ 1 or with documented peripheral arterial disease) 7 who were unsuitable for vitamin K antagonist therapy. The trial included 5,599 patients, of whom 2,808 received apixaban in a blinded, double-dummy design. Patients who met two of the three following criteria—age ≥80 years, body weight ≤60 kg, or serum creatinine ≥133 µmol/L (1.5 mg/dL)—received a study dose of 2.5 mg apixaban twice daily, while all others received 5 mg twice daily. To distinguish thromboembolic events from bleeding events in our analyses, stroke included ischemic and unspecified stroke but excluded hemorrhagic stroke, and vascular death excluded fatal bleeding. Bleeding was defined according to the definition of International Society on Thrombosis and Haemostasis (ISTH). 8 All outcome events were adjudicated by a committee blinded to the treatment arm. Compliance was estimated using pill counts which were performed at each study visit. Estimated glomerular filtration rate (eGFR) was calculated using the CKD-EPI formula. 9 The trial was stopped early after a median follow-up of 1.1 years due to clear evidence of superiority of apixaban.

Follow-up visits were scheduled at 1 and 3 months after randomization and every 3 months thereafter. Blood samples for this study were collected at the 3-month visit. Patients were asked to hold the dose of study drug preceding the visit so that the blood sample was taken prior to the morning dose. Citrated blood was double spun to produce platelet poor plasma, aliquoted, and centrally stored at −80°C for future analyses. Apixaban has previously been shown to remain stable at −70°C for up to 2 years, 10 and all samples were tested within this time frame.

The AVERROES trial was approved by all appropriate regulatory authorities and ethics committees (ClinicalTrials.gov NCT004969769). All patients provided written informed consent before study entry.

### Measurement of Plasma Apixaban-Calibrated Anti-FXa Activity

Anti-Xa activity was measured by a chromogenic anti-factor Xa assay (Rotachrom Heparin, Diagnostica Stago S.A.S, Asnières-sur-Seine, France). Since apixaban calibrators were not commercially available at the time the assays were done, apixaban calibrators were prepared by crushing apixaban tablets and dissolving in DMSO, as per Barrett et al. 10 Results were calibrated against a standard curve developed from these calibrators (500–15.6 ng/mL). The standard curve remained linear down to 15.6 ng/mL, which was therefore considered as the lower limit of quantitation of the assay. Values below this limit were transposed to 15.6 ng/mL for the purpose of statistical analysis. All calibrators and patient samples were measured on a STA-Compact instrument (Diagnostica Stago S.A.S.). The anti-Xa assays were performed by Hemostasis Reference Laboratories (Hamilton, Ontario, Canada).

### Statistical Analysis

Incidence of clinical outcomes is expressed per 100 patient-years. Comparisons between apixaban dose groups (5-mg twice daily and 2.5-twice daily) were performed using Wilcoxon's rank-sum test for continuous variables and by chi-squared test for categorical variables. Associations between outcomes and anti-Xa activity were determined using a Cox proportional hazards model. For the graphical display of these associations, logistic regression was used to estimate the probability of an event for each anti-Xa activity value. Analyses of the association between anti-Xa activity and clinical variables were restricted to patients receiving the 5-mg twice-daily dose. Correlations between anti-Xa activity and continuous variables were calculated by Spearman's correlation, while associations between anti-Xa activity and binary variables were examined by Wilcoxon's rank-sum test. Independent predictors of anti-Xa levels were determined by stepwise multiple linear regression with backward elimination. Only patients with available compliance data were included in the compliance analyses. Compliance rates for those in the lowest anti-Xa activity decile were compared against those in the upper nine deciles by Wilcoxon's rank-sum test. All analyses were performed using SAS version 9.4 software (SAS Institute, Clary, North Carolina, United States).

## Results

### Clinical Characteristics of Study Population

Blood samples were available for 2,392 (85%) of 2,808 participants randomized to apixaban, of whom 2,247 received the 5-mg twice-daily dose and 145 received the 2.5-mg twice-daily dose. Clinical characteristics of tested patients are presented in Table 1 . Patients receiving the 2.5-mg twice-daily dose were older, had lower body mass, had lower eGFR, were more likely to be female, had higher CHADS _2_ score, and were less likely to be taking a moderate or strong inhibitor of P-glycoprotein (Pgp) or CYP3A4 than those receiving the 5-mg twice-daily dose ( *p* < 0.05 for each comparison; Table 1 ).

**Table 1:** Apixaban anti-Xa activity (ng/mL) and clinical characteristics for the entire group and according to apixaban dose

Apixaban dose	Either dose ( *n* = 2,392)	2.5 mg BID ( *n* = 145)	5 mg BID ( *n* = 2,247)	*p* -Value
Median anti-Xa activity (ng/mL)	122 (63–198)	99 (60–146)	125 (64–202)	0.003
Age (y)	70 (63–76)	84 (81–86)	69 (62–75)	<0.001
Weight (kg)	78 (66–91)	56 (50–60)	79 (68–92)	<0.001
eGFR (mL/min/1.73 m ^2^ )	71 (54–92)	38 (32–46)	73 (57–94)	<0.001
Female sex	969 (41%)	95 (66%)	874 (39%)	<0.001
History of heart failure	954 (40%)	56 (39%)	898 (40%)	0.75
CHADS _2_ score 0–1	869 (36.3%)	14 (10%)	855 (38%)	
CHADS _2_ score 2	896 (37.5%)	62 (43%)	834 (37%)	<0.001
CHADS _2_ score >2	626 (26.2%)	69 (48%)	557 (25%)	
Concomitant use of CYP3A4 or Pgp inhibitor (moderate or strong)	553 (23.2%)	23 (15.9%)	530 (22.6%)	0.032
Liver disease (baseline elevation of bilirubin, AST, or ALT)	145 (6.1%)	8 (5.5%)	137 (6.1%)	0.78

Abbreviations: ALT, alanine aminotransferase; AST, aspartate aminotransferase; CYP3A4, cytochrome P450 3A4; eGFR, estimated glomerular filtration rate; Pgp, P-glycoprotein.

Notes: Values presented as median (interquartile range) or number (%). *p* -Value is for comparison between doses by Wilcoxon's rank-sum for continuous variables and chi-squared test for categorical variables. Analysis by concomitant use of a CYP3A4 or Pgp inducer (moderate or strong) is not included due to small numbers ( *n* = 5).

### Apixaban-Calibrated Anti-FXa Activity

**Fig. 1:**
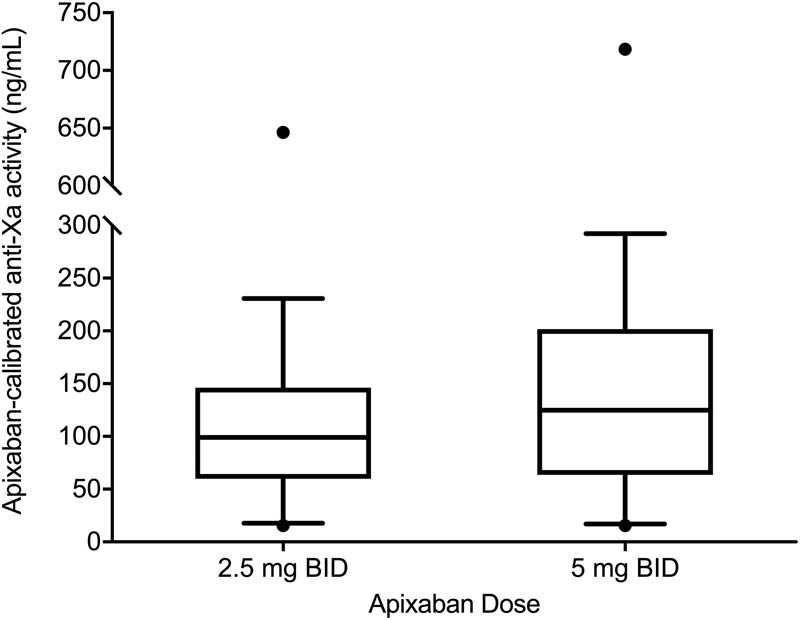
Apixaban-calibrated anti-Xa activity by dose. Blood samples were drawn immediately prior to the morning dose of apixaban. Boxes represent median and interquartile range, whiskers represent 10th to 90th centiles, and points represent minimum and maximum.

The median anti-Xa activity was 122 ng/mL (interquartile range [IQR]: 63–198 ng/mL) and the interindividual geometric coefficient of variation (GCV) was 113%. In 230 (9.7%) of the patients, the anti-Xa activity was below the lower limit of quantitation of the calibrated anti-factor Xa assay (15.6 ng/mL). The 10th to 90th centile range was 17 to 289 ng/mL. Patients receiving the 2.5-mg twice-daily dose had a 21% lower median anti-Xa activity compared with those receiving the 5-mg twice-daily dose (99 ng/mL, IQR: 60–146, 10th to 90th centile range: 18–231 vs. 125 ng/mL, IQR: 64–202, 10th to 90th centile range: 17–292; *p* = 0.003 for comparison of medians; Fig. 1 ).

### Anti-Xa Activity and Clinical Outcomes

**Fig. 2:**
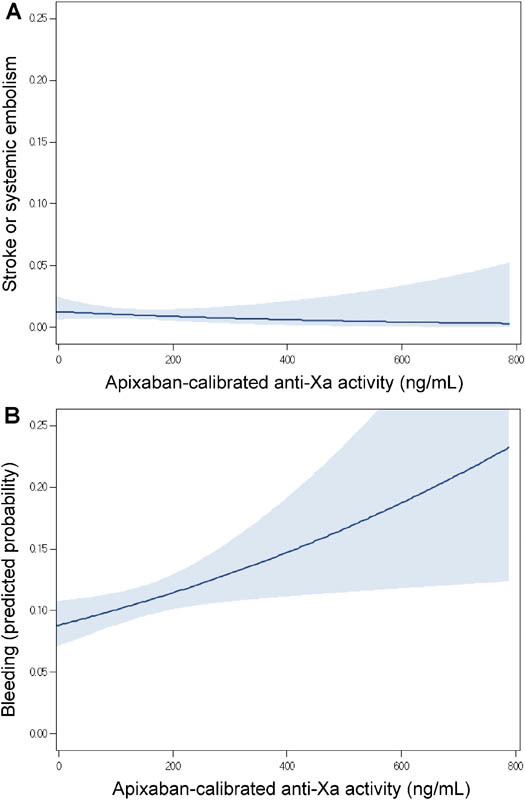
Stroke/systemic embolism and bleeding versus anti-Xa activity. Predicted probability of ( **A** ) stroke/systemic embolism; or ( **B** ) bleeding of any severity (minor, clinically relevant nonmajor, or major) versus anti-Xa activity. The shaded region represents the 95% confidence interval.

The rates of clinical outcomes according to quintile of anti-Xa activity are presented in Table 2 . No significant association was found between anti-Xa activity and stroke/systemic embolism ( Fig. 2A ), myocardial infarction, or symptomatic pulmonary embolism ( *p* ≥ 0.44 for each), although event rates were low, limiting power to detect a relationship. In a post hoc analysis, patients within the lowest decile of anti-Xa activity had a significantly greater risk of stroke than those with higher anti-Xa activity (Fisher's exact test, *p* = 0.013). There was an association between anti-Xa activity and occurrence of bleeding (of any severity; *p* = 0.01 by Cox regression; Fig. 2B ). This was driven mainly by minor bleeding ( *p* = 0.009 by Cox regression), with rates of 3.55, 6.92, 6.34, 7.39, and 8.19 per 100 patient-years for the lowest to highest quintiles, respectively, of anti-Xa activity. The rates of major bleeding were low, and there was no detected association between anti-Xa activity and major bleeding in a Cox regression analysis ( *p* = 0.581). Similarly, no association was detected with clinically relevant nonmajor bleeding ( *p* = 0.621 by Cox regression; Table 2 ).

**Table 2:** Clinical events by apixaban anti-Xa activity quintile

	Quintile 1	Quintile 2	Quintile 3	Quintile 4	Quintile 5	*p* -Value (Cox-regression)
Range of anti-Xa activity: ng/mL	0–50	51–98	99–146	147–254	255–719	
Embolic or thrombotic events: *n* (/100 patient-years)						
Ischemic/unspecified stroke or systemic embolism	10 (1.82)	2 (0.37)	2 (0.38)	6 (1.08)	4 (0.76)	0.44
PE	0 (0)	2 (0.37)	2 (0.38)	0 (0)	0 (0)	0.55
MI	5 (0.91)	2 (0.37)	3 (0.57)	6 (1.08)	1 (0.19)	0.50
Bleeding events: *n* (/100 patient-years)						
Any bleeding	35 (6.68)	50 (9.82)	51 (10.33)	61 (11.84)	59 (11.94)	0.01
Major bleeding	6 (1.09)	5 (0.91)	11 (2.12)	5 (0.90)	7 (1.33)	0.58
Clinically relevant nonmajor bleeding	12 (2.20)	15 (2.80)	12 (2.33)	20 (3.67)	14 (2.68)	0.62
Minor bleeding	19 (3.55)	36 (6.92)	32 (6.34)	39 (7.39)	41 (8.19)	<0.01
GI bleeding	3 (0.54)	1 (0.18)	3 (0.58)	0 (0)	3 (0.57)	1.00
Death: *n* (/100 patient-years)						
Total death	14 (2.53)	14 (2.55)	14 (2.67)	14 (2.50)	16 (3.02)	0.23
Vascular death	9 (1.62)	9 (1.64)	12 (2.29)	11 (1.97)	14 (2.64)	0.05
Fatal bleeding	0	0	2 (0.382)	0	1 (0.189)	0.50

Abbreviations: GI, gastrointestinal; MI, myocardial infarction; PE, pulmonary embolism.

Notes: Values for outcomes represent total number of events (events/hundred patient-years). *p* -Value is for correlation between outcome and apixaban anti-Xa activity in a Cox regression model.

### Effect of Clinical Characteristics on Anti-Xa Activity

We evaluated the clinical predictors of trough apixaban anti-Xa activity in the 2,247 patients who received the 5-mg twice-daily dose. Increasing age correlated with increased anti-Xa activity ( *p* < 0.001). Female sex, history of diabetes, and use of a CYP3A4 or Pgp inhibitor (moderate or strong) were also associated with higher anti-Xa activity ( *p* < 0.001, *p* = 0.011, and *p* = 0.002, respectively). Body weight and eGFR were negatively correlated with anti-Xa activity ( *p* = 0.002 and *p* < 0.001, respectively), and history of heart failure was associated with lower anti-Xa activity ( *p* < 0.001; Table 3 ). In the stepwise multiple linear regression model, eGFR, female sex, history of heart failure, body weight, age, and use of moderate or strong inhibitors of CYP3A4 or Pgp remained independent significant determinants of anti-Xa activity ( *p* < 0.05).

**Table 3:** Effect of clinical characteristics on apixaban anti-Xa activity

Characteristic	Subgroup 1	Subgroup 2	Subgroup 3	Subgroup 4	*p* -Value
Age (y)	<65 ( *n* = 737)	65 to <80 ( *n* = 1,268)	≥80 ( *n* = 242)	–	
Anti-Xa activity	101 (47–168)	131 (71–208)	166 (107–255)	–	<0.001
Weight (kg)	>100 ( *n* = 321)	>60 to 100 ( *n* = 1,668)	≤60 ( *n* = 226)	–	
Anti-Xa activity	115 (65–167)	126 (65–204)	135 (46–274)	–	0.002
Sex	Male ( *n* = 1,373)	Female ( *n* = 874)	–	–	
Anti-Xa activity	115 (61–183)	141 (70–232)	–	–	<0.001
CHADS _2_	0–1 ( *n* = 855)	2 ( *n* = 834)	>2 ( *n* = 557)	–	
Anti-Xa activity	121 (68–188)	119 (57–204)	136 (64–224)	–	0.066
Heart failure	Yes ( *n* = 898)	No ( *n* = 1,348)	–	–	
Anti-Xa activity	105 (48–194)	133 (75–207)	–	–	<0.001
eGFR (mL/min/1.73 m ^2^ )	≥80 ( *n* = 931)	50 to <80 ( *n* = 962)	30 to <50 ( *n* = 317)	<30 ( *n* = 17)	
Anti-Xa activity	105 (53–163)	133 (71–212)	177 (86–294)	274 (136–424)	<0.001
Diabetes	Yes ( *n* = 433)	No ( *n* = 1,813)	–	–	
Anti-Xa activity	134 (79–208)	122 (61–199)	–	–	0.011
Concomitant use of CYP3A4 or Pgp inhibitor (moderate or strong)	Yes ( *n* = 530)	No ( *n* = 1,717)	–	–	
Anti-Xa activity	141 (68–225)	119 (63–194)	–	–	0.002
Liver disease (baseline elevation of bilirubin, AST, or ALT)	Yes ( *n* = 137)	No ( *n* = 2,110)	–	–	
Anti-Xa activity	118 (56–185)	125 (64–203)	–	–	0.261

Abbreviations: ALT, alanine aminotransferase; AST, aspartate aminotransferase; eGFR, estimated glomerular filtration rate.

Notes: Analysis is for patients receiving 5-mg twice-daily dose only. Anti-Xa activity is median apixaban-calibrated anti-FXa activity in ng/mL (interquartile range). *p* -Value is for Spearman's correlation for continuous variables and Wilcoxon's rank-sum test for binary variables. Analysis by concomitant use of a CYP3A4 or Pgp inducer (moderate or strong) is not included due to small numbers ( *n* = 5).

### Compliance Analyses

A compliance analysis was performed in 2,062 patients (86%) with available compliance data. Compliance was lower in patients in the lowest decile of anti-Xa activity compared with the upper nine deciles (94.6%, IQR: 80.4–98.6% vs. 96.5%, IQR: 90.3–99.1%; *p* < 0.001 by Wilcoxon's rank-sum test). When including only those patients with ≥80% compliance ( *n* = 1822), anti-Xa activity was no longer positively correlated to risk of minor bleeding by Cox regression ( *p* = 0.212). The difference in risk of stroke between patients within the lowest decile of anti-Xa activity and those with higher anti-Xa activity was no longer statistically significant (Fisher's exact test, *p* = 0.070).

## Discussion

We performed this substudy of AVERROES to examine three items: (1) the range of on-treatment trough levels; (2) the relationship between trough levels and clinical events; and (3) clinical predictors influencing trough levels in AF patients taking apixaban. Our findings either extend or provide new information on all three.

### Range of On-Treatment Levels

Previous data on the range of usual on-treatment levels in AF patients treated with apixaban were limited to predicted range from pharmacokinetic–pharmacodynamic modeling or to small studies. 11 12 13 Our study provides more accurate and precise estimates than those obtained from predicted models or from small studies, respectively, because samples were taken from a much larger population of AF patients ( *n* = 2,392) taking apixaban. The range of levels found in our study is in keeping with the interpatient variability in drug levels observed with other direct oral anticoagulants. 14 15 Patients receiving the reduced dose of 2.5-mg twice-daily had approximately 20% lower median and 90th centile of anti-Xa activity as those receiving 5-mg twice-daily, but the lowest decile of both groups was very similar ( Fig. 1 ). Since the clinical criteria for reduced dose apixaban were chosen because they had been shown to be associated with increased drug activity as well as an increased risk of bleeding, 16 the current dosing recommendations seem to have appropriately resulted in decreasing the proportion of patients with high drug levels in this group, without increasing the proportion with the lowest levels. Our observations support the use of the currently employed clinical criteria for dose reduction. A previously published registry reported that a significant proportion of patients taking apixaban for AF were prescribed the 2.5-mg twice-daily dose when they did not meet criteria for dose reduction as specified in AVERROES and ARISTOTLE. 17 Our data do not provide direct information on the drug levels or risk of clinical events in such patients.

### Relationship between Trough Level and Clinical Events

Based on the RE-LY (dabigatran) and ENGAGE AF-TIMI 48 (ENGAGE-AF, edoxaban) trials, 14 15 we expected to find relationships between apixaban trough anti-Xa activity and the risk of both major bleeding and of stroke/SEE. In both the RE-LY and ENGAGE-AF subanalyses, the risk of major bleeding increased with trough level, but an increased risk of stroke/SEE was observed only when levels fell into low extremes. We observed a significant relationship between trough levels and total bleeding, but not with major or clinically relevant bleeding. This may be explained by the fact that both RE-LY and ENGAGE-AF had considerably more events (>10 times the number of embolic and bleeding events in ENGAGE-AF compared with AVERROES), and therefore power to detect relationships. The lack of strict timing of blood sampling in our study, the inclusion of events before and after blood sampling, and the use of anti-Xa activity rather than liquid chromatography with tandem mass spectrometry may also have reduced our ability to detect such relationships. However, our finding that the relationship between stroke/SEE and levels was observed only when trough apixaban anti-Xa activity fell below the lowest decile is consistent with the findings reported in RE-LY and ENGAGE-AF. About 90% of patients in the lowest decile had anti-Xa activity below the level of detection, of whom 24% had compliance less than 80%. We cannot rule out the possibility that the relationships seen for minor bleeding and stroke with anti-Xa activity were driven by patients with absent drug levels due to noncompliance.

### Clinical Predictors of Trough Levels

In an analysis restricted to patients taking 5 mg BID, we found renal function, age, sex, body weight, diabetes, heart failure, and use of inhibitors of CYP3A4 or P-gp to be independent clinical predictors of trough apixaban levels. Of these seven clinical factors, renal function had the most important influence on apixaban levels, followed by age, sex, and weight. As shown in Table 3 , as eGFR varied from ≥ 80 to ≤ 30 mL/min, median trough apixaban level increased more than 2.5-fold from 105 to 274 ng/mL. This finding was unexpected because in selecting patients taking 5 mg BID, we excluded those who had extreme values for three recognized criteria (age ≥ 80 years, weight <60 kg, and serum creatinine > 1.5 mg/dL) of an increased response to apixaban. Such a finding could be important clinically because it raises the possibility that the currently accepted clinical criteria for dose reduction could be improved by including either eGFR ≤ 30 mL/min as an independent criterion for dose reduction or by developing a weighted score system based on the identified clinical predictors (renal function, age, sex, body weight). The small number of patients taking the 2.5-mg twice-daily dose limited our ability to separately analyze this subgroup.

Our findings indicate that apixaban is effective and safe across a broad range of anti-Xa levels and provides no support for routine laboratory monitoring. Knowledge of usual on-treatment drug levels should also be helpful when managing patients taking one or more drugs that interact with apixaban; high or low apixaban levels in this situation could prompt clinicians to change drug therapy to ensure that patients remain effectively and safely protected against the risk of stroke.
